# Health diplomacy to promote multisectoral participation in fighting against fragmentation and increasing budget for internalization of the health financing progress matrix in Burundi

**DOI:** 10.1186/s13561-022-00376-w

**Published:** 2022-06-02

**Authors:** Alexandre Nimubona

**Affiliations:** Université Sagesse d’Afrique & Ministry of Public Health, Bujumbura, Burundi

**Keywords:** Health diplomacy, Health financing progress matrix, Multisectoral collaboration, De-universalization, Internalization, Health gap, Health gradient, Universal health coverage, Proportionate universalism

## Abstract

**Background:**

Regardless of its form, financing health in isolation will never raise sufficient funds to lead to universal health coverage. Achieving this goal which is not a pure health policy, requires multisectoral collaboration to support financing mechanisms. Within this framework, the World Health Organization has created the Health Financing Progress Matrix to assess a country’s progress in health financing. The World Health Organization calls for multisectoral support for health financing systems to achieve universal health coverage. This paper aims to explain how health diplomacy can be defined and implemented to influence and facilitate multisectoral participation in fighting against fragmentation and increase necessary budget to internalize the health financing progress matrix in Burundi.

**Main text:**

Burundi’s health financing system is characterized by multiple fragmentation of resources and services, which reinforces economic and health inequities, referred to as de-universalization of universal health coverage. The health financing system in Burundi is inadequate to meet the health needs of the population. Different people with different needs form different segments, and coverage may be inconsistent, duplicative, or incomplete. Health diplomacy can alleviate this situation by appointing health finance attachés in each of the 19 sectors that make up the life of the country. Health finance attachés may have three main tasks:1) promoting confidence building, 2) seeking consensus, and 3) building solidarity for universal health coverage. The practices of health finance attachés can help to improve budget for more coverage. Following the World Health Organization’s progress matrix on health financing, internalization can be achieved in four ways: (i) raising the profile of health diplomats to be accredited in non-health sectors, (ii) establishing offices of health finance attachés in each sector, (iii) creating means by which sectors benefiting from internalization act, (iv) operationalizing proportionate universal health coverage.

**Conclusion:**

Health diplomacy holds an ethical practice (representation approach) for internalizing the matrix. Measuring the size of the health gap and the steepness of the health gradient determines the degree of matrix internalization. Health diplomacy needs to be included in all health financing agendas to achieve proportionate universal health coverage in poor countries like Burundi.

## Background

In current discussions, scientific researchers agree that, regardless of its form, financing health in isolation will not raise sufficient funds to lead to Universal Health Coverage (UHC). The reason is simple – a decade and a half after the promulgation of UHC [1], at least 50% of the population still lack access to the basic health services they need, 36% of available health services are financially unaffordable [2], and about 53% of the world’s population has financial difficulties to pay for their health services [3]. Current health financing policies remain inconsistent to achieve full coverage of the population, services, and costs globally [4].

In low-income countries, national/domestic financing is insufficient to achieve UHC [5, 6]. As a fungible and volatile asset that countries cannot rely on, external financing will also be insufficient [7]. Since many structural (income, education, employment, gender, distance, age) [8, 9] and social (politics and culture) [10] determinants of UHC lie outside the health sector, even diverse sector-wide financing, i.e., well-coordinated and decently pooled sector financing will never bring about full coverage.

In sub-Sahara African countries, the capacity and accessibility of UHC services is only 27%, and 58% of these services are not financially affordable [2]. Financing the health sector as an isolated and closed system cannot achieve the no pure health goal of UHC [11]. It is therefore credible that the degree of UHC achievement depends on the linkages between health and the other sectors in financing – for example, supporting financing through multisectoral collaboration has resulted into 53% of health services being accessible in Rwanda by increasing population coverage at more than 83% through community-based health insurances and contracting some private health facilities for primary healthcare services. Population coverage enhanced patient visits at primary healthcare level to 1.94 per capita per year versus 1 visit recommended in resource-limited countries by the World Health Organization (WHO) [2] – in 2019, Rwanda has already met some key requirements and is now the most advanced country in sub-Saharan Africa regarding UHC [12].

To guide health financing policy development and practices, particularly in countries with low progress on UHC like Burundi, the WHO has created a standardized assessment tool called the “Health Financing Progress Matrix (HFPM)” [13] as a common point of reference to drive progress towards UHC. Table 1 presents a summary description of this matrix.

**Table 1:** The WHO matrix description

The WHO matrix contains guiding principles expressed in the form of seven assessment areas and nineteen desirable attributes both summarized here in four main components: • Multisectoral collaboration in financing, • Improving predictable and stable budget through financial and non-financial support, • Ensuring coordination of funds to prevent, avoid or alleviate fragmentation, and • Providing timely financial data to inform priority services to be improved.	
The purpose of this health financing assessment tool at country level, is to provide annual evidence-based feedback to policy makers about gaps and enhancements to be made to accelerate progress on UHC. The WHO matrix recommends country assessment findings to be stated in the form of four progress levels:	
Emerging	No clear policy that guides implementation or practices
Progressing	There is policy under development with some aspects being implemented
Established	There is approuved policy which is being implemented, assessed, and regularly adjusted to comply with the international standards
Advanced	There is approuved policy aligned with the international standards, disseminated in all sectors, effectivelly implemented nationally, and with systematic annual assessment to inform policy design improvements and implementation.
With this progress matrix, the WHO has launched a call for multisectoral participation, such as bringing all stakeholders to the table, assessing together, closing gaps together, and jointly creating a national progress matrix [14] to build a decent UHC structure. Avoiding fragmentation, raising and maintaining sufficient budget for health are the main focus of the WHO matrix implementation at a country level.	

The preparation of the country matrix requires a return to multisectoral collaboration recommended in the 1978 Alma-Ata Declaration (Article 4) [15] to improve or maintain predictable and coordinated budget for health. However, there is no specification about how can this multisectoral collaboration, be implemented and measured to inform progress levels defined in the WHO matrix. The general framework of this paper is thus to set out the importance of health diplomacy in building and measuring multisectoral participation when internalizing the matrix in Burundi. More specifically, the paper aims to explain how health diplomacy can be defined and implemented to influence and facilitate multisectoral participation in fighting against fragmentation and increase necessary budget to internalize the health financing progress matrix in Burundi, and collaboratively achieve UHC, where no sector is left behind.

This article is the first to address this issue. Before concluding, the main text seeks to contextualize the importance of internalizing the matrix, argues fragmentation as one of the causes of limited progresses in Burundi, and maps out channels for the WHO matrix internalization across the country.

### Why should the matrix be internalized?

The Health Financing Progress Matrix is more relevant to Burundi as it provides common international evidence-based benchmarks that the country can follow to make progress on health financing indicators. By following this matrix, the country will seek to meet international health financing standards in its way, according to local needs and context. This process, whereby the country develops its health financing system to meet the nineteen common features of the matrix, is referred to here as internalization. This paper uses the ideas of Renous, L. R [16], to argue that internalization of the WHO matrix in Burundi should consist of aligning the country with the matrix attributes (summarized into four components see in Table 1) in designing and implementing health financing reforms to make progress towards UHC. While internalizing the matrix requires multiple actors and multisectoral collaboration [17], it now seems appropriate to find a way to peacefully promote multisectoral engagement in building a health financing system (country matrix) aligned with the WHO matrix in Burundi, a country with rather limited UHC performance.

Burundi is a small, poor, and landlocked country in East Africa (more than 67% of Burundians live in extreme poverty [18], i.e. on less than USD 1.9 per day). The country is still lagging – its overall progress towards UHC is slowing [19] or declining (de-universalized) and therefore unacceptable due to fragmentation of its health financing system.

In Burundi, the gap between UHC services provided to the population and the recommended best UHC services (gap to UHC) varies between 57% [2] and 60% [20].

The following section highlights two forms of fragmentation at the cause of this limited progress in Burundi.

### Fragmentation as cause of limited progress

Burundi is suffering from the process of declining or decreasing coverage, commonly termed de-universalization [21] of UHC, due to two forms of fragmentation: (i) medicalization of the health sector that features fragmentation in decision making, where health is seen as a purely medical service, and (ii) fragmentation of the existing restricted budget. Medicalization involves the use of medical language and models to identify and solve health issues [22, 23]. Evidence of this is that more than 76.9% of clinical doctors employed in the public sector hold managerial positions [24]. The negative outcome of this non-multisectoral approach, which leaves non-medical skills behind in the management of the health sector, is the exodus of good clinical doctors, moving the country 90% away from the UHC standard (0.1 doctors per 10,000 inhabitants instead of 1/10000). As a result, people are not always cared for by providers who have the appropriate clinical skills. This affects quality by widening the gap between the need for medical care [25] and the medical care provided [26](care gap).

Management positions can be given to non-medically trained people like economists, health services managers, … to maintain sufficient clinical doctors in health facilities and the reasonable level of medical care quality.

The poorer quality resulting from low regulation of the health system has paved the way for more health inequities in Burundi. Some Latin American countries with inequitable [27] and corrupted [28] health systems, such as Mexico, show that the low regulation of a decentralized health system, like that of Burundi [29, 30] with different capacities of decentralized units, promotes fragmentation in decision making and increases health inequities [31]. In Burundi, these inequities that arose from fragmentation in decision making are exacerbated by fragmentation in financial coverage [32, 33] (see Table 2).

**Table 2:** Structural fragmentation of financial coverage

	Vertical fragment 1	Vertical fragment 2	Vertical fragment 3
	**Non-contributory approaches:** *From taxes, prices, and donors; *For formal and informal people; *Covering institutional functioning and vertical diseases programs	**Voluntarily contributory approaches:** *From payroll deductions and individual contributions; *For informal people, private sector formal workers, and geographical areas; *Covering specific and limited set of services.	**Compulsorily contributory approaches:** * From payroll deductions; * For civil servants and security bodies; *Covering a specific and limited set of services.
**Horizontal fragment 1**: output based-payment mechanisms	Medical Assistance Card	Medical assistance card	National Office for Pensions and Occupational Risks
	Performance-Based Financing and Free Healthcare	Community-based health insurances (more than 125 micro-insurance)	National Institute of Social Security
	Fee exemption for needy people	Private health insurances (more than 10)	Public Service Health Insurance
	Foreign aid	Out-of-pocket payments	
**Horizontal fragment 2**: input based-payment mechanisms	Government’s share		
	Foreign aid		

Source: Adapted from [29, 32],

A closer look at this table shows that the health system in Burundi consists of three vertical fragments of financial coverage based on contributory and non-contributory approaches. Each vertical fragment contains separate segments for specific funding conditions (type of facility, drugs, etc.).

At the horizontal level, Burundi’s health financing system includes two fragments based on output and input financing mechanisms. Both vertical and horizontal fragmentation of financing disrupts the universal nature of health services. Health service provision is fragmented – services are delivered according to people’s characteristics and in an uncoordinated way, while they must of course be aligned with needs.

Based on the targeting of services (e.g. essential medicines for children under five within performance-based financing and free healthcare), the fragmentation of health service provision, applied between diseases or similar, makes comprehensive care impossible and promotes social segregation.

For example, 35.5% of sick pregnant women do not receive the free health care they need, and 23.6% of sick children under 5 years of age do not receive the health care they need [34], as per the policy of performance-based financing and free health care. This example shows that those who receive free health care first belong to the better-off classes. That said, some needy people are pushed out of the free healthcare system in favor of the better-off – this unfair practice to needy people will be difficult to correct. Burundi can learn from the experiences of some Latin American countries, which are always worth considering, as they show that it is easy to introduce segmentation and complex to undo it [35, 36]. For example, Chile and Colombia introduced segmentation voluntarily in the 1990s, resulting in huge health disparities. Chile took more than two decades to correct this into four ways: (i) addressing management issues of the health sector that resulted in the effective functioning of the network of services within different geographical areas, (ii) budget control, (iii) increasing additional resources in the form of consumer tax, (iv) defining prioritized set of services guaranteed to all, and (v) ceiling user fees threshold at 20% of the reference price. In sum, Chile developed a comprehensive rights-based health system that includes all segments of the population into a universal system which resulted into significance progress level on UHC [37]. Colombia never did – despite improvements made in national health spending (mandatory health insurance, increased budget allocated to health, …) equality of healthcare utilization remains a big challenge [38].

Based on the ability to pay, population segmentation has increased liberalization and commercialization of health service provision, resulting in the proliferation of small private health facilities and over-the-counter pharmacies competing with each other. For example, the cost of the same service provided by the same doctor varies from one health facility to another in the private sector, and the same medicine is priced differently from one pharmacy to another. Such commercialization leads to mechanisms that discriminate between the haves and the have-nots. For example, 17% of the sick do not have access to the health services they need [18] and 81.5% of patients have to make catastrophic or impoverishing payments [39]. These negative effects of co-payments put Burundi’s health financing system (segregated financing) on the path of UHC de-universalization, which promotes inequalities between people’s income and service delivery [21].

What does generic progress matrix for health financing look like in this situation? Burundi’s health financing system, characterized by parallel schemes with poor fundraising mechanisms and weak coordination of funds, is still at the level of emerging progress for three main reasons that are at odds with the UHC ethic expressed in the trilogy of goals and objectives of the health financing progress matrix – three final goals (quality-financial security-utilization of services by those who need them) and three intermediate objectives (transparency-equity-efficiency) [14].

- **First**, Burundi’s health financing system has divided the population into four categories: (i) civil servants with specific financial coverage; (ii) pregnant women and children under five with specific financial coverage; (iii) employed and rich with private financial coverage; and (iv) the poorest, employed and unemployed without financial coverage.
- **Second**, such segmentation of the population has created two main segments for health services – public and private.
- **Third**, the share of user fees varies between health financing and health service segments (heterogeneity of user fees varies from 0 to 100%), leading to under-fragmentation of funds and services.

In this context, coverage may become de-universalized, i.e. it becomes inconsistent, duplicative, or incomplete, highlighting the need for a national progress matrix.

The next section explains how can the health diplomacy pave the way for mitigating fragmentation and enable more progress on UHC.

### Curbing fragmentation by health diplomacy

Alleviating fragmentation needs to go beyond the health sector and abroad – this requires health diplomacy practices ranging from multisectoral and multi-institutional expertise to support for financing mechanisms [40]. Health diplomacy is a nascent discipline for which there is no single definition [41]–in the present context, it refers to “the way of gaining trust to promote multisectoral collaboration for a particular health intervention” [42, 43]. The paper uses the only consensual and common concept of “representation approach” argued in all definitions of health diplomacy [44] to characterize and measure multisectoral participation to work towards two outcome areas mapped by nineteen desirable attributes and seven technical assessment areas of the WHO matrix [13, 14], such as (i) closing the gap in health status between healthy and unhealthy people (health gap), and (ii) closing the gap in economic status between the haves and the have-nots (health gradient) [45].

Health finance attachés, created to operationalize the representation approach [44] in each sector from which funds for health should come, are well suited to promote and generate more coordinated resources for health in all sectors [46]. To mitigate the threat of fragmentation, health finance attachés are expected to apply three key ethical practices, [43] in their respective home sectors to which they should pay more attention in order to build coherence of financing schemes, improve collaboratively the outcomes of the national health financing progress matrix and shape together the path towards UHC:

1. **Increasing confidence** in health services by regularly informing the public about the quality of UHC services available (e.g. how funds are raised and used) through media, Faith-based groups, posters, corporate visits, non-state actors, conferences, … should help to improve knowledge and evidence, one of the ethical practices also suggested by the WHO matrix [13]. This appropriate health-related diplomatic practice to make the whole population aware of the quality of services available will draw the attention of health service providers to promote the quality of health services for all. For example, promoting the quality of primary health care in Costa Rica has increased confidence in a successful, progressive health financing system in which the poorest 20% of the population benefits from 30% of health spending while promoting the quality of obstetric care in Brazil has narrowed the gap in antenatal care (difference between antenatal care provided and required), with a narrowing of the gap between the poorest 40% and the rich 40% [31]. In addition, the consultation processes and the intensity of close multisectoral collaboration are intended to build some confidence [43] in the country assessment reports that inform decision making about priority interventions to form a unified or coordinated health financing system among all stakeholders locally involved in financing.
2. **Seeking consensus** (through continuous multisectoral negotiations initiated by health finance attachés) on key strategic directions for internalizing the WHO matrix should increase the priority given to health [47] and also raise unanimously more attention to budget allocation in the other social sub-sectors that can directly improve sustainably progress on UHC, such as housing and food security for example.
3. **Build solidarity** between health and non-health professions to mitigate cross-sectoral risks in health financing. Cross-sectoral solidarity built by health finance attachés for an integrated network of health services, as is the case in the health systems of Brazil, Cuba, and Costa Rica [31], will obviously limit fragmentation.

Using these diplomatic health practices could further align Burundi’s health financing system with the WHO matrix standards (matrix internalization) for a decent UHC. But in what way? The following paragraphs attempt to provide an answer.

### Recommended ways to internalize the WHO matrix

The internalization of the matrix can take place in four successive ways:

- **One**. Build a profile of health sector representatives (or health diplomats, or health finance attachés) to be accredited in non-health sectors across the country. The experience in Pakistan [48] indicates two ways in which Burundi can train this profile to strengthen its diplomatic capacity in the health sector: (i) in the short term, through on-the-job training and mentored experiences for health workers selected according to need; (ii) in the long term, through the development of an academic program for health workers preparing for a career of health diplomat. Integrating the health financing progress matrix [14] into all health training programs is the right way to internalize it.
- **Two.** Establishing health finance attachés offices in each sector [48].
- **Three.** Identify means by which sectors internalizing the WHO matrix should raise necessary health budget. The paper identifies 19 internalization target sectors [49] in Burundi (see Table 3)

**Table 3:** Menu of recommended ways for the WHO matrix internalization in each sector

	Receiving sectors	Recommended ways to internalize the WHO matrix nationwide
1	Agriculture	Introduce mechanisms for taxing non-agricultural property based on the size of the non-agricultural property. For example, taxation of USD 0.005 per area per year should encourage people to use their landholdings, increase land revenue [50] and household income, and thus improve their economic situation.
2	Breeding	Increase taxes on the sale of domestic animals to keep enough domestic animals and get more organic fertilizer on the land. More organic fertilizer should increase farm income – this should improve individual economic status [51]and in turn help improve individual health status.
3	Peach	Introduce an annual boat registration system to increase annual taxation on boats and watercraft – this should help increase tax revenue [52] dedicated to health.
4	Trade	Increasing purchase taxes on non-essential consumer goods such as jewelry, more expensive clothing, electronic devices, etc. is intended to reduce disposable personal income to encourage people to buy less of these non-essential goods. In this sense, only people with higher economic status will consume non-essential goods and give a job. The health gradient will remain at a certain level which will enable economic growth [53].
5	Mining	Raising taxes on mining production, import, and export to an optimal level will ensure that the mining sector contributes significantly to the maximum reduction of the health gradient between citizens and society as a whole [54] at the national level.
6	Industry	Expansion of the progressive industrial tax system, where rich industries are taxed more than poor ones (because their ability to pay increases as their income increases). This progressive tax system aims to distribute income equitably [55] in order to reduce inequalities in economic status.
7	Energy	Introduce a tax on fuel at the pump - levy a tax on the amount of fuel purchased at the pump (e.g. 0.5 USD per liter consumed) to raise public revenue [56] to be used for health care and to achieve equity goals. The introduction of a tax on electricity bills (with an exemption for people of low economic status) should also generate much-needed revenue for vulnerable groups [57] and reduce the health gap.
8	Transport	The introduction of a tax on airline tickets for all flights out of the country is a consistent source of revenue for heath [58]. Some kind of transport tax to be paid at the beginning of the fiscal year (e.g. in July of each year as the cut-off date) by every citizen who wants to travel around the country by bicycle, tricycle, motorbike or car is also a sustainable source of revenue for health.
9	Justice	Introduce a legalized and regulated model of sin and luxury taxes from six main areas: (i) tobacco use, (ii) alcohol and cannabis use, (iii) sugary drinks, (iv) gambling, (v) sex work [59]; and (vi) luxury cars. These types of health taxes [60] are intended to limit the consumption of luxury goods and services that harm the individual economy and hurt personal health status
10	Security	Dividend for health – reducing defense spending and reallocating funds to health is intended to help create and maintain peace, the only indicator of economic growth [61].
11	Infrastructure	Increase the share of property tax – an annual property tax should lead to a successful valuation of land and be the nation’s own predictable, efficient and equitable revenue source [62]
12	Religion	Organize annual crowdfunding for health – a channel for fundraising to finance health through awareness-raising campaigns [63, 64] in churches. Everyone should make his financial contribution an act of charity.
13	Public administration	(i) Strengthen mechanisms for cost control, planning, pricing, and budgeting for health [13]; (ii) introduce payroll deductions for health among workers from all sectors; (iii) increase administrative taxes. All these measures should help to promote equity [65] and increase the efficiency of resource allocation.
14	Communication	Taxing mobile phone bills and internet – less than 5% tax on mobile phone bills should generate more tax revenue [66] that can be used for health.
15	Tourism	Develop tourism sites and infrastructure – one tourist room creates 2.5 jobs [67], sources of payroll deductions, and taxes that can be allocated to health.
16	Health	Improve multisectoral coordination of health financing and networking of health services, expanding services coverage of quality
17	Education	Introduce a system of contribution vouchers for health – proof of payment of a certain amount (e.g. 1% of school fees) by each student at the beginning of each school year (with an exemption for the have-nots).
18	Bank and insurances	Establish a national bank specializing in banking for health services. E.g. currency creation for health services purchase (interest income), tax incentives for returns, and the like. The government should grant the bank all power and legal authority to provide financial solutions for the health services and to regularly provide innovative financing for health.
19	International cooperation	To link external funds with national revenues as recommended in the Paris declaration [68].

Source: Created from the literature review

This table might lead one to believe that:

(i) The internalization of the health financing progress matrix should focus more on multisectoral revenue generation, which is the weakest function of health financing in Burundi, according to the requirements of the Country Assessment Guide [14].
(ii) Improving the current tax system, i.e. collecting additional taxes from ordinary taxpayers, is a fundamental way [69] to build the country matrix, as this will help the country raise more funds for health to reach the UHC standard (USD 112 per capita per year). The internalization of the progress matrix for health financing faces the challenge of designing the structure and level of taxes to reduce corruption and embezzlement [70] – the internalization of the matrix is to design tax policies for health in all sectors at a level that does not discourage economic activity but encourages the change of undesirable behaviors across the country (the country matrix oriented tax policy). The study by Aaron REEVES and his collaborators offers profound insights worth considering when internalizing the WHO matrix in Burundi – a dollar more in tax revenue per capita yields about a tenth of the additional public health spending per capita in low-income countries. Tax financing is a fundamental way to ensure access to quality health services for all, everywhere [69]. Improving tax revenues from multiple sectors increases resources for UHC and discourages some health-damaging behaviours [71].
(iii) Each sector must continually find new ways to raise revenue to make fundraising a dynamic, complementary market among all the 19 sectors that make up the life of the country to increase the predictability of funds and reliance on domestic progressive revenue sources as long as the matrix standards recommends [14].
(iv) The establishment of a national health financing bank with the dual role of pooling the collected funds and purchasing health services is one of the expert solutions to internalize the WHO matrix. The bank is intended to serve as a strategic tool for good coordination of cash flows and strategic purchasing of health services, both of which are proposed by the matrix standards [13]to meet the needs of the population. The bank is to use its profits to repay the waiver of taxes from the have-nots. The Bank should also serve as an independent and general governor of pooling for the implementation and evaluation of the country matrix.

- **Four**. Operationalize proportional universal health coverage, where the main focus of resource mobilization is to reduce both the steepness of the health gradient and the size of the health gap [45] – resources are allocated proportionally to the needs of the population, regardless of their ability to finance them. Following the principle of “from each according to his income to each according to his need”, proportionate universal health coverage aims to make both health services and health funds accessible to all groups of the population, but with a relative intensity that corresponds to the level of economic and health status [72].

How? As a rule, populations with little or no ability to finance health services have high need for health services – these populations, who tend to be the poorest and have lower economic and health status, require a high dose of universal interventions (i.e. a high dose of services and purchase). Conversely, populations with a higher capacity to finance health services have a low need for health services – these populations, who are generally the wealthiest and have a higher economic and health status, require a low dose of universal interventions. All this means that, in general, as the capacity to fund health services and economic status increases, health status increases and the need for health services decreases, justifying a low dose of universal interventions, and vice versa.

But this is not always automatically the case. Some populations with higher economic status have poorer health status (e.g. the rich with chronic diseases) and have high need for health services, which requires a higher dose of universal interventions. There are also populations with lower economic status, but with better health status, who need fewer health services, indicating a lower dose of universal interventions.

Achieving the goal of proportionate universal health coverage requires individualization of health services and their acquisition (individual dosage of universal interventions). Proportionate universal health coverage requires the same interventions for all but at different intensities, which is referred to as dosing of universal interventions based on individual needs [73], to mitigate fragmentation and prevent the phenomenon of de-universalization of UHC.

## Conclusion

It is important to bear in mind that any UHC policy is in practice universal to all. Some degree of inequality is still required to maintain the momentum of the UHC policies. The internalization of the health financing progress matrix should be used to define the level of inequality that should be targeted. In Burundi, the targeted universal health coverage, the most emblematic and labor market objective of the current health financing system, has created a false sense of universalism due to the lack of a reference level for inequalities. The targeted coverage has led to division of some population groups, i.e. dividing the population into different groups to be covered (e.g. civil servants, children under five, pregnant women, and others) and dividing the UHC policy into staggered projects (e.g. the performance-based financing and free health care policy had four consecutive project stages from 2010 to date). In this respect, it is more unfair to think of UHC as uniformity for the entire population (with different needs). These findings give little hope that internalization of the health financing progress matrix will be easily achieved unless all sectors that make up the life of the country are articulated in a complementary manner and within a single national framework for building a decent UHC. Health diplomacy holds an ethical practice (representation approach) to enable and measure this multisectoral complementarity. While the matrix can be internalized in many different ways (country-specific), this ethical practice is intended to make health diplomacy one of the channels to avoid fragmentation and increase financial resources required to internalize the WHO matrix, which in turn is seen as a starting point for proportionate universal health coverage across the country. Not surprisingly, health diplomacy has not been cultivated among health professionals, which means that health care delivery remains inadequate to reduce both the health gap and the health gradient – the two expected outcomes of the WHO matrix internalization. By measuring the size of the health gap and the steepness of the health gradient in a country, we can determine the degree of internalization of the WHO matrix for progress in health financing. Health diplomacy needs to be included in all health financing agendas to achieve proportionate universal health coverage in poor countries like Burundi.

## Data Availability

Not applicable.

## Abbreviations

UHC: Universal Health Coverage
HFPM: Health Financing Progress Matrix
WHO: World Health Organization
